# Federated learning enables intelligent reflecting surface in fog-cloud enabled cellular network

**DOI:** 10.7717/peerj-cs.758

**Published:** 2021-11-22

**Authors:** Abdullah Lakhan, Mazin Abed Mohammed, Seifedine Kadry, Karrar Hameed Abdulkareem, Fahad Taha AL-Dhief, Ching-Hsien Hsu

**Affiliations:** 1 College of Computer Science and Artificial Intelligence, Wenzhou University, Wenzhou, China; 2 College of Computer Science and Information Technology, University of Anbar, Ramadi, Iraq; 3 Noroff University College, Kristiansand, Norway; 4 College of Agriculture, Al-Muthanna University, Samawah, Iraq; 5 Faculty of Engineering, School of Electrical Engineering, UniversitiTeknologi Malaysia (UTM), Johor Bahru, Malaysia; 6 Department of Computer Science and Information Engineering, Asia University, Taiwan; 7 Guangdong-Hong Kong-Macao Joint Laboratory for Intelligent Micro-Nano Optoelectronic Technology, School of Mathematics and Big Data, Foshan University, Foshan, China; 8 Department of Medical Research, China Medical University Hospital, China Medical University, Taiwan

**Keywords:** IRSTS, Offloading, ML, Objectives, Energy, Delay

## Abstract

The intelligent reflecting surface (IRS) is a ground-breaking technology that can boost the efficiency of wireless data transmission systems. Specifically, the wireless signal transmitting environment is reconfigured by adjusting a large number of small reflecting units simultaneously. Therefore, intelligent reflecting surface (IRS) has been suggested as a possible solution for improving several aspects of future wireless communication. However, individual nodes are empowered in IRS, but decisions and learning of data are still made by the centralized node in the IRS mechanism. Whereas, in previous works, the problem of energy-efficient and delayed awareness learning IRS-assisted communications has been largely overlooked. The federated learning aware Intelligent Reconfigurable Surface Task Scheduling schemes (FL-IRSTS) algorithm is proposed in this paper to achieve high-speed communication with energy and delay efficient offloading and scheduling. The training of models is divided into different nodes. Therefore, the trained model will decide the IRSTS configuration that best meets the goals in terms of communication rate. Multiple local models trained with the local healthcare fog-cloud network for each workload using federated learning (FL) to generate a global model. Then, each trained model shared its initial configuration with the global model for the next training round. Each application’s healthcare data is handled and processed locally during the training process. Simulation results show that the proposed algorithm’s achievable rate output can effectively approach centralized machine learning (ML) while meeting the study’s energy and delay objectives.

## Introduction

The usage of healthcare sensors has been growing in network capacity and ubiquitous wireless networking recognition to numerous leading enabling technologies such as the ultra-dense network (Chen et al., 2021). The quarry of 100 billion healthcare sensors devices by the upcoming fifth-generation (5G) wireless network has been widely met (UDN), Huge multi-input, multi-output system (MIMO) and communication using millimetre waves (mmWave) (Kousiouris et al., 2019). For the healthcare system, the distributed fog cloud implemented at different communication networks. However, the high complexity and hardware costs and the increased energy demand are still open questions. Densely installing base stations (BSs) or access points (APs) in a UDN, for example, not only increases hardware and maintenance costs but also exacerbates the network interference issue. Extending large MIMO from sub-6 GHz to mm Wave frequency bands is also typically expensive (Zhao et al., 2020). As a result, advanced, spectral, and energy-efficient but cost-effective solutions for future/beyond-5G wireless networks still needed for the healthcare sensors network (Qu et al., 2020).

The FL-IRSTS system can be intellectuals controlled by a cleverly reflecting surface (IRS) to extend flag concentrated obtained at the goal. This is often in stark differentiate to past procedures that made strides in remote communications by tweaking the sender or recipient. An IRS made up of a few IRS units, each of which can speak to the occurrence flag at a diverse point. The remote flag voyages from the source to the IRS optimized the goal in such IRS-assisted communications. Such a communication approach is precious when there’s a weak remote channel between the origin and destination due to boundaries or destitute natural conditions, or when they don’t have a clear line of location (Zhou et al., 2020). The IRS anticipated playing a noteworthy part in 6G systems due to their capacity to design remote situations, agreeing to numerous remote communications specialists. NTT DoCoMo and MetaWave, a startup in Japan, illustrated the utilize of IRS-like innovation for helping remote communications within the 28 GHz band. The expansive MIMO innovation utilized in 5G communications compared to IRS reflects remote signals. Hence, employments less control, whereas tremendous MIMO transmits signals and needs a parcel more control (Saha, Misra & Deb, 2020).

The computational offloading of IoT healthcare sensors, combined with IRS-assisted communications, enables a fog-cloud network in this paper. In the context of IRS-assisted wireless communications, we address the issue of Internet of Things healthcare sensors offloading computation tasks to a base station fitted with fog nodes. IoT healthcare sensors in the study plan to send computation tasks to the base station through wireless channels, with an IRS assisting communication between them. As compared to the art of studies, the study has the following contributions.

• The study devises a new IRS that enables the energy-efficient and cost-efficient distributed system for healthcare applications in distributed fog cloud. The proposed system is different than (Saha, Misra & Deb, 2020; Hosseinalipour et al., 2020) studies where these studies proposed IRS aware energy-efficient computational offloading for healthcare applications. However, this study considered a more complex communication system as compared to existing studies.

• The study first creates an algorithm framework to detect feasibility when given an arbitrary set of information rate constraints to solve the energy and cost-efficient computational offloading problem. When we determine that the problem is feasible, the algorithm will also have a possible solution. Then we devise a solution that maximizes the earning potential of fog cloud nodes.

• The importance of IRS devices to gain significant numerical performance. According to the performance assessment and results from the discussion, IRS will substantially improve the feasibility of distributed healthcare fog-cloud networks for information rate constraints. Furthermore, numerical results show that our algorithm converges quickly and can significantly boost fog-cloud nodes to achieve energy and cost results.

The rest of the paper designs as follows. “Related Work” expresses the related work of studies to the task scheduling problem. “Problem Description” illustrates the problem under research works establishes it. The proposed algorithm framework and its components discussed in “Proposed FL-IRSTS Strategy Framework”. The performance evaluation of algorithms defined in “Performance Evaluation and Results”. Conclusion and future work of the paper enclosed in “Conclusion and Future Work”.

## Related Work

In this session, the manuscript discussed the existing efforts in intelligent reflecting surface (IRS) for applications in terms of computational offloading in terms of computation and communication technologies. The main problem to be solved based on machine learning and federated learning. Furthermore, joint technologies such as fog-cloud computing and communication technology by intelligent reflecting surface (IRS) can give many advantages in practice to the sensor devices and healthcare applications. The related work part studies the efforts of both fog-cloud computational offloading and communication technology in IRS. Whereas, energy-efficient fog-cloud aware computational offloading aware framework suggested in Chen et al. (2021), Kousiouris et al. (2019), Zhao et al. (2020), Qu et al. (2020). In many studies Zhou et al. (2020), Saha, Misra & Deb (2020), fog-cloud paradigm aware system suggested to minimize end to end latency of healthcare applications. The works (Hosseinalipour et al., 2020; Sharma, Park & Cho, 2020) analyze a network with a large number of IoT sensors users and a single base station with a single fog node. By comparing the obtained utilities under the two conditions, mobile devices choose computation offloading to edge cloud or local computing on the devices. Both network communications use the same wireless channel and conflict with one another. The dynamics of offloading IoT healthcare sensor data offloading decision-making modelled as a game, then analyzed using future game theory. Instead of just one radio, discuss a wireless obstacle setting with several channels in Lakhan et al. (2021b).

Table 1 shows existing objectives, methods, centralized machine schemes, federated learning techniques and different offloading and scheduling suggestions in the literature work. These studies trained the mobility model of application in adaptive environment based on machine learning unsupervised learning. The single parameters enabled (Single-para) static model based on supervised learning widely implemented in existing IRS system. The goal was minimized the overall load balancing issues of the network. Whereas, multiple parameters enabled training model based on unsupervised without labeling for IRS system widely investigated in the defined studies in the table. The data uploading rate of a single healthcare sensor uses computation offloading compared to the data uploading rate of other sensors on the same wireless channel of the fog-enable have many base-stations aware system devised in Lakhan et al. (2021c). The goal is to minimize the energy efficiency of the sensors in the network. The work (Lakhan & Li, 2020) addresses the computation offloading of a power system to a fog node, where the addition of energy-efficient communicating nodes and complicates the computation offloading policy design. The latency and energy-efficient aware computation offloading for edge computing in MIMO the multicell scheme studied recently. The goal is to transmit precoding matrices of mobile devices, as well as computational resources assigned by the edge cloud to the devices, which are included in the optimization variables to minimize mobile devices’ energy consumption (Mujeeb-ur Rehman et al., 2021). IRS-aided communications have recently received a lot of attention in the literature. IRS support transmissions from IoT sensors to base stations because our work also focuses on uplinks since IRSs can manage to represent incident wireless signals in the desired way. In Lakhan et al. (2021a), Chen, Liu & Peng (2019), Lakhan & Xiaoping (2018) investigate the effect of channel estimation errors on uplink data speeds as defined above. In addition to the uplink studies mentioned above, downlinks examined in Lakhan & Li (2019) for IRS assisting communications between mobile devices and base stations. The fact that there seem to be more downlink studies than uplink studies may explain that the base station will centralize transmit beamforming optimization in the former case. The direct communications between healthcare sensors and IRS widely improve the communication capacity during offloading and downloading.

**Table 1:** Existing IRS methods and systems.

Research	Parameters	Decision	Training	Environment	Methods	Objective
Chen et al. (2021)	Single Para.	Static	Network	Loss Function	IRS	Min. Energy
Kousiouris et al. (2019)	Single Para.	Static	Program	Distributed	IRS	Min. Energy
Zhao et al. (2020)	Single Para.	Static	REST API	Centralized	IRS	Min Computation
Qu et al. (2020)	Two Para.	Dynamic	RPC	ML	Centralized	Max. Utilization
Zhou et al. (2020)	Multi-Para.	Dynamic	Monitoring	Adaptive	Centralized	Max. Throughput
Saha, Misra & Deb (2020)	Multi-Para.	Dynamic	Resource	Adaptive	Centralized	Min. Delay
Hosseinalipour et al. (2020), Hosseinalipour et al. (2020), Sharma, Park & Cho (2020)	Multi-Para.	Dynamic	Monitoring	Adaptive	Centralized	Min. Energy
Lakhan et al. (2021b), Lakhan et al. (2021c)	Multi-Para	Hybrid	Monitoring	Mobility	Centralized	Min Rent
Lakhan & Li (2020), Mujeeb-ur Rehman et al. (2021), Lakhan et al. (2021a)	Many-Para.	Hybrid	SDN-Controller	Mobility	Centralized	Min. Cost
Chen, Liu & Peng (2019), Lakhan & Xiaoping (2018)	Many-Para.	Hybrid	SDN-Controller	Mobility	Centralized	Min. Budget
Lakhan & Li (2019)	Many-Para.	Hybrid	OS	Mobility	Centralized	Min. renting cost
Proposed Work	Energy/Latency	Fog-Nodes	Federated learning	Energy/Latency	Node Learning	Global decision

To our knowledge, no research has been done on the energy and delay-effectiveness of IoT healthcare sensors in distributed fog-cloud networks with IRS. Existing studies used the IRS in the fog computing system to solve the latency minimization problem. This paper proposes a novel IRS for a fog-cloud-assisted healthcare sensor system, in which the cost and energy efficiency of nodes are optimized.

## Problem Description

**Figure 1:**
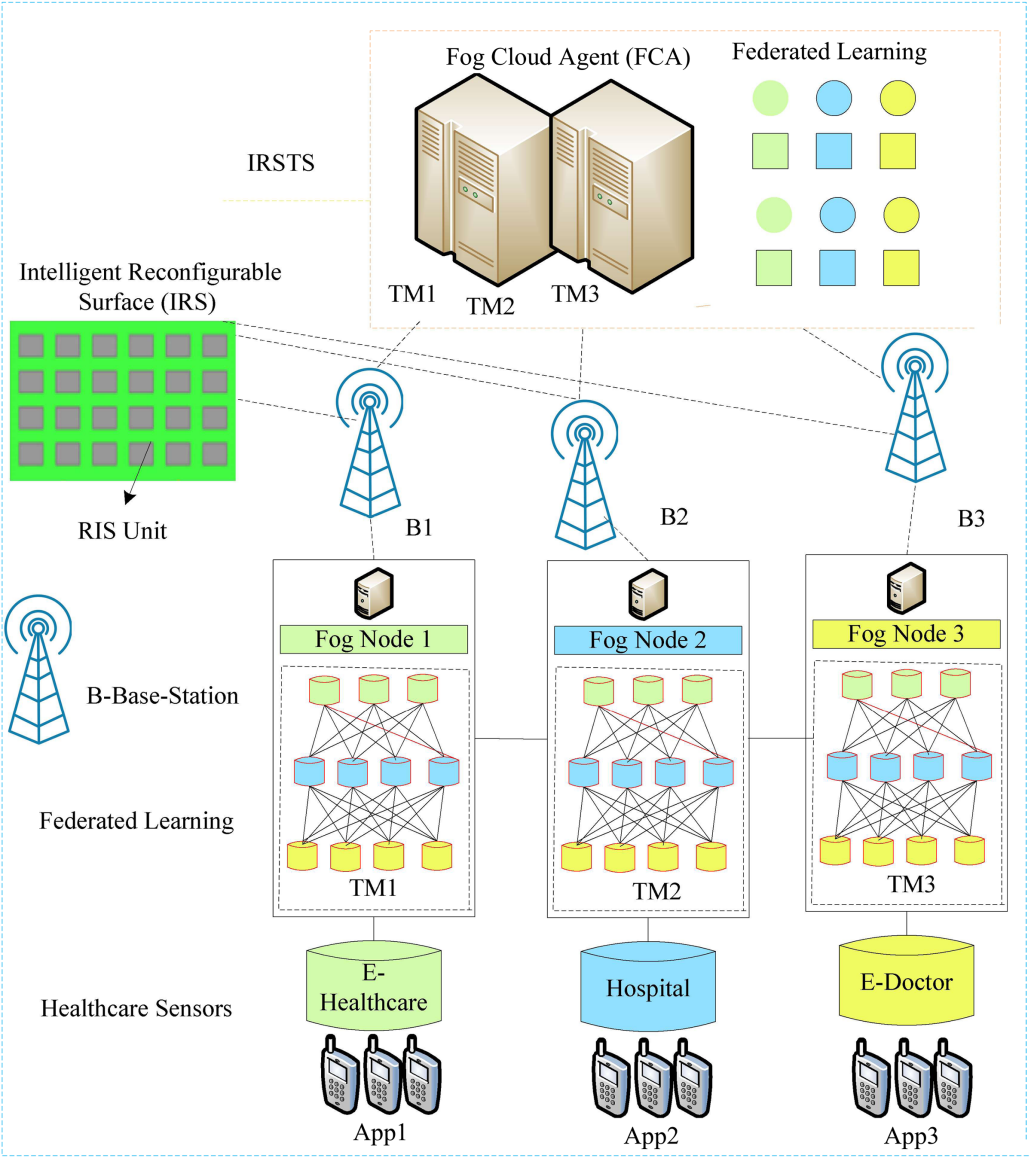
IRS-enable federated learning aware system.

The study devises novel federated learning awareness, and IRS enable base-station assisted of fog nodes at the user the network as shown in Fig. 1. The system consists of different components which are accountable for doing the entire process in the network. The user applications (app) are healthcare and connected to the proximity fog-enable base stations. All the base stations configured by the IRS controller in the cellular network with the one-kilometre range. The fog cloud agent (FCA) manages all the fog-nodes controller in the system.

### Applications

The applications are coarse-grained and offload the entire workload to the nearby ubiquitous fog-enable base stations for processing the network. The system has ubiquitous connectivity. Therefore, IRS can configure many base stations which are connecting for mobile communication for healthcare applications.

### Fog-enable distributed base-stations

In Fig. 1, the fog nodes are sub-data centres of cloud computing implemented as the edge nodes at the different base stations. The IRS monitors and schedules the uplink and downlink requests and results of applications in other base stations at the runtime. At the same time, all fog nodes are heterogeneous by their configuration. Each fog node train the model of offloaded data for further processing in the system. The training is the federated learning in the design, where FCA executes the workload decision based on achieved distributed federated learning data from different nodes. Each base station has limited space, range and energy band for processing the requests in the system. The IRS must handle the energy and performances of nodes in the ubiquitous and mobile environment.

### Federated learning

Federated learning concedes learning models to be distributed through devices and systems that perform initial training and then send modified model attributes to centralized cloud servers for stable and train data on supervised learning based on genetic algorithm aggregation and global model creation. This way, the decision and learning ratio becomes a high and high performance to the applications. All the local training models of healthcare data at fog-nodes shared with the global training model in FCA for further processing.

### Problem formulation

The study considered the *I* number of healthcare sensor coarse-grained applications, *i.e*., $\{ i = 1, \ldots ,I\}$. The *W*_*i*_ is the workload of *i*^*th*^ application. The Notation *D*_*i*_ shows the deadline of application *i*. The *B* represents the number of base-stations, *i.e*., $\{ {B_1}, \ldots ,B\}$. The Notation *B*_*C*_ denotes the capacity of the particular base station during the offloading and downloading of data between node and sensors. The study consider the *K* number of computing nodes, *e.g*., $\{ {k_1}, \ldots ,K\}$. All computing nodes are different in their configuration, such as lateness, energy, memory. Each node contains the *S* number of virtual machines. The virtual machine *s*_*k*_ belongs to the particular node *k*. Each node has limited resources and speed, represented by respective notations, *e.g*., *ε*_*k*_ and *ζ*_*k*_. All notations description can find in Table 2.

**Table 2:** Mathematical notation.

Notations	Descriptions
*I*	Number of IIoT applications
*i*	*i*^*th*^ application of *I*
*D*_*i*_	The deadline of *i*^*th*^ app.
*W*_*i*_	The Workload of application *i*
$T_i^e$	Execution delay of app. *i*
*PC*_*i*_	Power consumption of app. *i*
*K*	Number of computing nodes
*k*	The *k*^*th*^ node of *K*
*k*_*P*_	The power consumption of *k*
*ε*_*k*_	The resource capacity of *k*
*ζ*_*k*_	The speed of node *k*
*E*_*k*_	Energy Power of node *k*
*L*_*k*_	Delay execution of node *k*
*B*	Number of Base-stations
*B*_*C*_	Resource capability of Base-stations *B*
*A*	Attributes of blockchain in block *B*
*TM*	Training model of any random node
*TM.P*_*W*_	Power consumption during model train
*x*_*isk*_	Assignment of workload

### Energy consumption

The system shown in Fig. 1 is ubiquitous and distributed with different nodes for healthcare applications. The energy model in IRS aware distributed base station is a bit complex. Therefore, the system measured the energy in various models in the system.

#### Energy consumption between sensors and base-station

**Figure 2:**
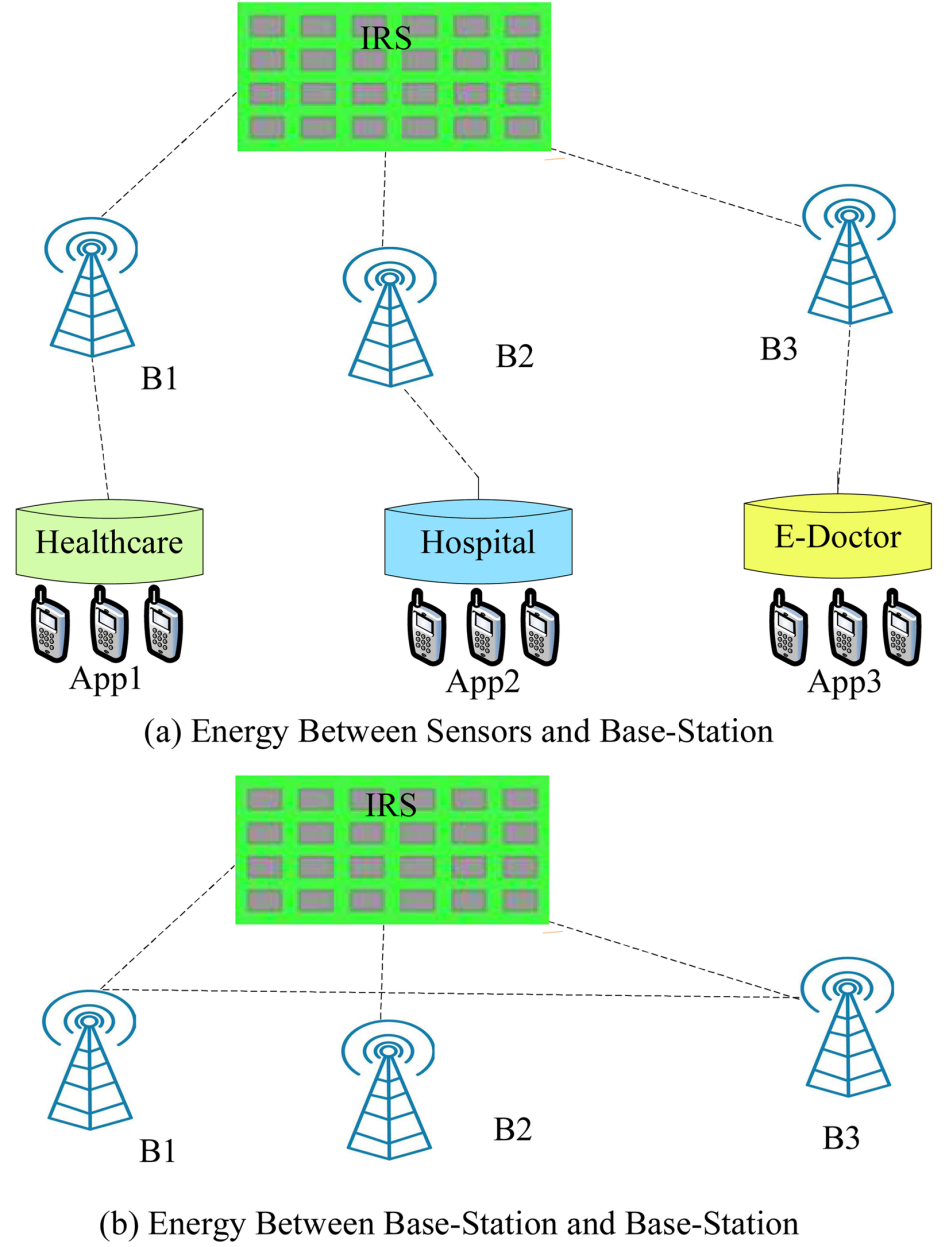
Energy consumption cases.

To comprehend the issues with cellular *B* power consumption, one must first investigate the design of these systems and the power consumption of their components. Access links connect mobile stations to a core network through *B*. As shown in Fig. 2A, these stations cover a cell divided into several sectors, with each *B* surrounded by a sector antenna. According to their coverage area, cellular *B* divided into macro-, micro Femto (indoor), and pico *B*, with each cell having its scale, output capacity, and data rate (Lakhan et al., 2021c). Because of their limited coverage range and low radiation power demand, small BSs use less power (Lakhan & Li, 2020; Mujeeb-ur Rehman et al., 2021; Lakhan et al., 2021a). As shown in Fig. 2B, a macro-BS site usually consists of many pieces of power-consuming equipment. The operational power of the macro-BS can model as follows.

(1) $$\matrix{ {{B_{Energy - 1}} = ({E_{Sect}} \times {E_{TX}})\displaystyle{{{P_{PA}} + {P_{BB}} + {P_{RF}}} \over {(1 - \lambda ES)(1 - \lambda DC)(1 - \lambda Cool)}}} \cr { + {P_{wm}} + {P_{au}}.} \cr }$$

In Eq. (1), *PA*, *BB*, *RF* implies the energy of the power amplifier (PA), the optical signal processing or baseband unit (BB), and the transceiver (RF). The outcome of *PA* is a linear function of *B* base station transmission energy *E*_*TX*_, and defined as $\textstyle{{{E_{TX}}} \over {PA}}$ and it represents *PA* power Efficiency. Where, 1 − *γES*, 1 − *γDC*, 1 − *γCool* represent the losses sustained by the rectifier, regulator, and active cooling, which are scaled linearly with the power consumption of the other components, respectively. *P*_*mw*_ stands for auxiliary equipment, *P*_*au*_ is a backhaul such as lighting and closed-circuit healthcare nodes and denotes the microwave backhaul connection. Since a BS has several sectors and antennas, the power consumption of these components must be multiplied by the number of base stations *E*_*Sect*_, and the *B* power consumption must be multiplied by the number of transmitting antennas *E*_*TX*_ for each sector.

#### Energy consumption between base-station to base-station

The model determines the energy-consumption of between base stations in the following way.

(2) $${B_{Energy - 1,2}} = \displaystyle{{{W_i}} \over {{B_w}}}$$

In Eq. (2), *B*_*w*_ is the available bandwidth between base stations to transfer the requests in the system.

(3) $$Mobility = BDensity\lambda \displaystyle{{{B^2}} \over {k{m^2}}}.$$

Eq. (3) determines the Energy efficiency (Mbit/Joule) between base stations during mobility of the workloads.

#### Sum of energy consumption

The proposed system consists of different computing nodes such as fog nodes and cloud node, where all nodes are heterogenous in their properties. Therefore, the power consumption directly proportional to the speed of the node during execution in the system. The high power node consumed much more power as compared lower power node. The study determined the power consumption of the node in the following way.

(4) $$\matrix{ {\vskip1.5pc}{E = \sum\limits_{i = 1}^I \sum\limits_{k = 1}^K \sum\limits_{s = 1}^S {x_{isk}}\displaystyle{{{W_i}} \over {{\zeta _k}}} \times T_i^e \times } \hfill \cr {{P_k} + BT.{P_W} + TM.{P_W} + {B_{Energy - 1}} + {B_{Energy - 1,2}}.} \hfill \cr }$$

The Eq. (4) determined the power consumption of resource during processing of applications in the system.

### Latency

The proposed system consists of different computing nodes such as fog nodes and cloud node. All nodes are heterogenous in their properties. Therefore, the execution speed of nodes depends upon memory and their properties. The high-speed node *k* has a lower delay as compared to the lower speed node *k*. The study determined the delay of applications in the following way.

(5) $$\tau = \sum\limits_{i = 1}^I \sum\limits_{k = 1}^K \sum\limits_{s = 1}^S {x_{isk}}\displaystyle{{{W_i}} \over {{\zeta _k}}} \times T_i^e + BT + TM.$$

The *τ* in Eq. (5) determined the total delay of all IIoT during execution in the system. All applications fully offloaded the entire coarse-grained workload to the system for further implementation. At the same time, the delay is the combination of execution delay $T_i^e$, base-station delay *BT*, and model training delay *TM*—the base-station delay calculated in the following way.

(6) $$BT = \sum\limits_{i = 1}^I \sum\limits_{k = 1}^K \sum\limits_{s = 1}^S \sum\limits_{{B_1} = 1}^B i.A.s.k.$$

The Eq. (6) calculated the base-station delay during offloading and downloading of sensor data. The training model time on data calculated in the following way.

(7) $$BT = \sum\limits_{i = 1}^I \sum\limits_{k = 1}^K \sum\limits_{TM = 1}^{TM} \sum\limits_{{B_1} = 1}^B i.A.TM.k \times {W_i}.$$

The Eq. (7) determined data training delay during processing in the system.

### Objective function

Multi-objective optimization, also known as Pareto optimization, is a branch of multiple criteria decision making that deals with statistical combinatorial optimization comprising various objective functions that must optimize at the same time. The study determined the objective function in the following.

(8) $$\min (f(\tau ),f(E)).$$

The Eq. (8) shows the multi-objective functions minimized optimization of processing application on different nodes.

Subject To

(9) $${\tau _i} = \le {D_i},\quad \forall \{ i = 1, \ldots ,I\} .$$

All applications must be executed under their deadline as defined in Eq. (9).

(10) $${W_i} = \le \varepsilon ,\quad \forall \{ i = 1, \ldots ,I\} .$$

All workload of applications must be less than the resource capacity of all nodes as determined in Eq. (10).

(11) $${x_{i,s,k,B,TM}},\quad \forall \{ i = 1, \ldots ,I\} .$$

Each workload exactly assigned to one node at a time as defined in Eq. (11).

(12) $${x_{i,s,k,B,TM}},\quad \forall \{ k = 1, \ldots ,K\} .$$

Each node exactly execute one workload at a time as defined in Eq. (11).

(13) $${W_i}.BT \le {B_C},\quad \forall \{ i = 1, \ldots ,I\} .$$

The Eq. (13) ensured that, all blocks can mined on limited transactions under their capacity.

(14) $${x_{i,s,k}} = \{ 0,1\} .$$

The workload can be assigned to one workload when *x*{*i*,*s*,*k*} becomes 1, otherwise it is equal to 0 as defined in Eq. (14).

## Proposed FL-IRSTS Strategy Framework

The study devises Federated Learning Intelligent Reconfigurable Surface Task Scheduling (FL-IRSTS) algorithm framework consists of different dynamic heuristics to solve the problem into sub-steps as shown in Algorithm 1. The algorithm consists of other heuristics to improve training, latency efficiency, and energy efficiency to solve the problem as shown in Algorithm 1.

**Algorithm 1:** FL-IRSTS algorithm framework

**Input:** $\{ i = 1, \ldots ,I\} ,\{ k = 1, \ldots ,K\}$;
**Output:** min τ, *E*;
1 **begin**
2 Call Initial Local Training to Global Training;
3 Initially schedules all workload based on their deadlines;
4 Call Algorithm 3;
5 Reschedule initial workloads to minimize the overall delay of applications;
6 Call Algorithm 4;
7 Reschedule initial workloads to minimise the overall energy consumption of nodes;
8 *i* ← *k* Optimize τ, *E*;
9 End-Loop;

**Algorithm 2:** Initial training of deadline sensor

**Input:** {i = 1, …, I}, {k = 1, …, K}, $\{ TM = 1, \ldots ,TM\}$
1 **begin**
2 Schedule-list [] = null;
3 **foreach** (*i* = 1 to *I*) **do**
4 Sort all workloads by their deadlines by Earliest Deadline First Method;
5 if ( $T_i^e \leftarrow k \le {D_i}$) **then**
6 Train initial model on possible node *TM* = *k* ←*W*_*i*_;
7 Initial Schedule all workloads based on their deadlines;
8 Add Schedule-list [*i* ←*k* ←*TM*];
9 End-Loop;

**Algorithm 3:** Lateness of sensor workloads

**Input:** *ζ*_*k*_, *ε*_*k*_, $\{ TM = 1, \ldots ,TM\}$, Schedule-list [*i* ← *k*];
1 **begin**
2 **foreach** (*Schedule-list* [*i* ← *k*]) **do**
3 Sort all computing nodes by their speed and resource capacity;
4 **if** (*ζ*_*k*_1 ≥ *ζ*_*k*_2 & *ε*_*k*_1 ≥ *ε*_*k*_2) **then**
5 Reschedule possible tasks from one node to another node;
6 Add Schedule-list [*i* ← *k*1 ← *TM* to *i* ← *k*2 ← *TM*];
7 optimize the objective function τ ← Schedule-list [*i* ← *k*1 ← *TM* to *i* ← *k*2 ← *TM*];
8 End-Loop;

Algorithm 1 is the framework which consists of different strategies as discussed in problem description and solve the problem in different steps. Each strategy to be explained in different sub-sections.

### Initial training of deadline sensor data

Algorithm 2 determines the initial training of senors workloads based on their deadlines based on the earliest deadline first scheme. The Schedule-list (*i* ← *k* ←*TM*) shows the list of initial scheduled which satisfied the quality of service of all workloads during scheduling in the fog-cloud node. The initial model will train based on the Convolutional neural network (Mujeeb-ur Rehman et al., 2021) method during the initial schedule. A convolutional neural network chooses a loss function while constructing and setting your model. There are a lot of loss functions to pick from, and it can be challenging to know which one to use, or even what a loss function is and what role it plays in neural network training. In work, the model will determine the correct loss function for your predictive modeling tasks, as well as the relevance of loss and loss functions in training deep learning neural networks.

### Latency scheduling

Algorithm 3 reschedule all initial tasks without missing deadline from lower speed node *k*_1_ to *k*_2_ if nodes have a free slot and enough space to continue the execution of workload from the previous node. The preemptive scheduling process where the running workload can switch from one node to another without degrading its quality of service during the process. The training of the model done at the initial scheduling time. Therefore, fast speed node can reduce the latency delay of applications during rescheduling.

### 4.3 Energy efficient IRS scheduling

Algorithm 4 determined the rescheduling of listed workloads from higher power node to lower power node during the processing of workloads without losing their deadline generosity. In this way, the algorithm can minimize the power consumption of all nodes and optimize the objective function *E*. Initially, Algorithm 4 determines the offloading plan based on Eq. (1). RIS chooses an optimal base station for uplink and downlinks the sensor requests in the system. However, if the requested base station *B* is fully loaded, RIS transfer the workload based on Eq. (2). The mobility of workload is handled based on Eq. (3).

**Algorithm 4:** Energy efficient task scheduling

**Input:** Schedule-list [*i* ← *k*1 ← *TM* to *i* ← *k*2 ← *TM*], $B = \{ B = 1, \ldots ,B\}$;
1 **begin**
2 **foreach** (*B* = 1 to *B*) **do**
3 **if** (*B*_*Energy*__−__1_) = *true* **then**
4 Minimize the offloading based on Eq. (4);
5 **if** (*B*_*Energy*__−__1,2_) = *true* **then**
6 Minimize the offloading based on Eq. (4);
7 **foreach** (*Schedule-list* [*i* ← *k*]) **do**
8 **if** (*mobility = true*) **then**
9 Optimize $Mobility = BDensity\lambda \displaystyle{{{B^2}} \over {k{m^2}}}$ based on Eq. (8);
10 Sort all computing nodes by their lower power consumption;
11 **if** (*PW*_*k*1_ ≥ *PW*_*k*2_ & *ε*_*k*_1 ≥ *ε*_*k*_2) **then**
12 Reschedule possible tasks from one node to another node;
13 Add Schedule-list [*i* ← *k*1 ← *TM* to *i* ← *k*2 ← *TM*];
14 optimize the objective function E ← Schedule-list [*i* ← *k*1 ← *TM* to *i* ← *k*2 ← *TM*];
15 End-Loop;

## Performance Evaluation and Results

This evaluation part conducted the experiments of healthcare workloads in the system. The existing approaches: Approach1 (Zhao et al. (2020); Saha, Misra & Deb, 2020; Hosseinalipour et al., 2020; Sharma, Park & Cho, 2020) and Approach2 (Kousiouris et al., 2019; Mujeeb-ur Rehman et al., 2021; Zhou et al., 2020) implemented that are nearly same structure as proposed scheme IRSTS framework in the system.

### Parameter setting

Table 3 defines the parameter setting of mathematical model in the simulator during experiments on the self-generated data healthcare applications in the system. The base-station energy calculated in joules per unit (jpu) per consumption and power per watt in the computing node as shown in Table 3.

**Table 3:** Simulation parameters.

Parameter	Value
*i* ∈ *I*	1,000 − *W*_*i*_ ← 1 *GB to* 20 *GB*
*i* ∈ *I*	*d*_*i*_ ←10 *to* 50 min
*B* = 1 ∈ *B*	*Bw* = 512 *mbps*, *E*_*Nsec*_ = 10, *E*_*TX*_ *jpu*
*B ← P*_*wm*_, *P*_*au*_	20, 30
*P*_*PA*_, *P*_*BB*_, *P*_*RF*_	12.4, 15.7, 11.33 jpu
*λ*_*ES*, *DC*, *Cool*_	29, 12, 7 jpu
*k*_1_ ∈ *K*	*ε*_*k*_, *k*_*p*_ 500 to 1,000 GB per 10 to 100 watt
Mobility	Density $\lambda \displaystyle{{50} \over 5}k{m^2}$

### Simulation environment

The simulation environment for federated learning Intelligent Reflecting Surface is Matlab-based open-source and available on Github. We merged these techniques in Ifogsim simulator (Chen, Liu & Peng, 2019) to design the modified research environment for the applications. The study implemented the Arduino wireless-based such as sound, vibration, pressure, widely exploited in the simulation to generate data. Furthermore, MOCH healthcare sensors kit (Chen, Liu & Peng, 2019) was implemented with the Ifogsim and analyzed the performances of applications with the proposed schemes in the system. In the Ifogsim, we added the convolutional neural network-based training by using the loss function. For instance, each application is deployed at sensors level or mobile, and the loss function train all data locally on mobile devices. Then the trained model of data offloaded to the fog cloud agent (FCA). The FCA in Ifogsim is a global model where all local train models of data are shared to all local devices of applications.

#### Observation of federated learning-IRS

**Figure 3:**
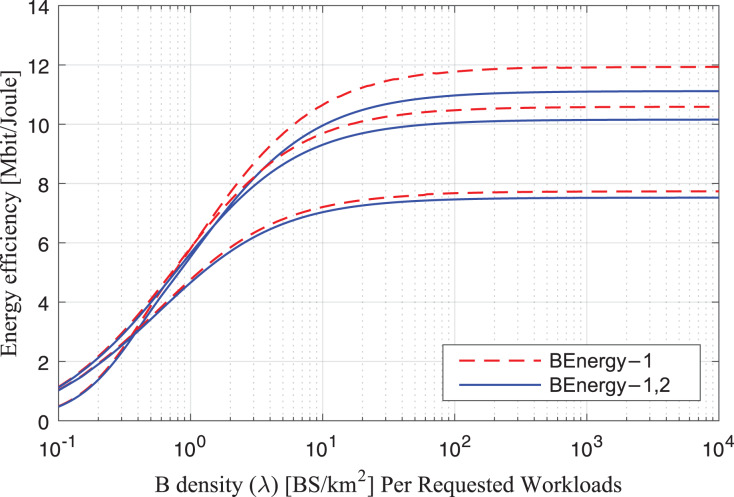
Energy consumption during requests.

**Figure 4:**
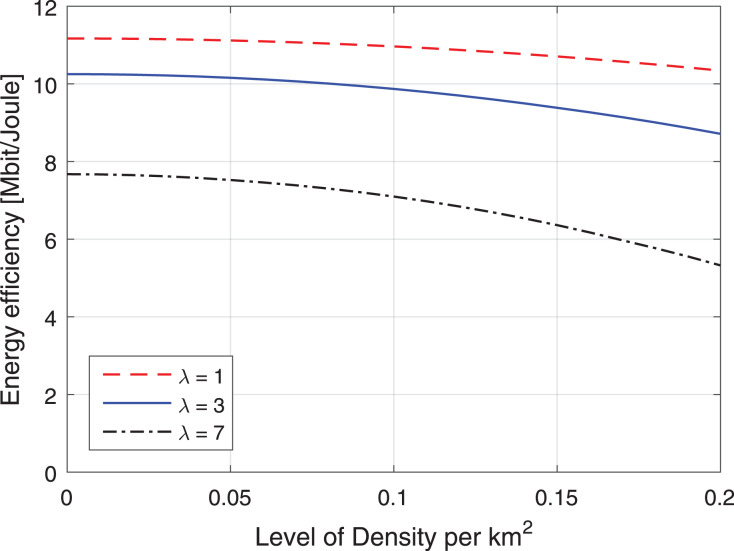
Energy consumption during requests.

**Figure 5:**
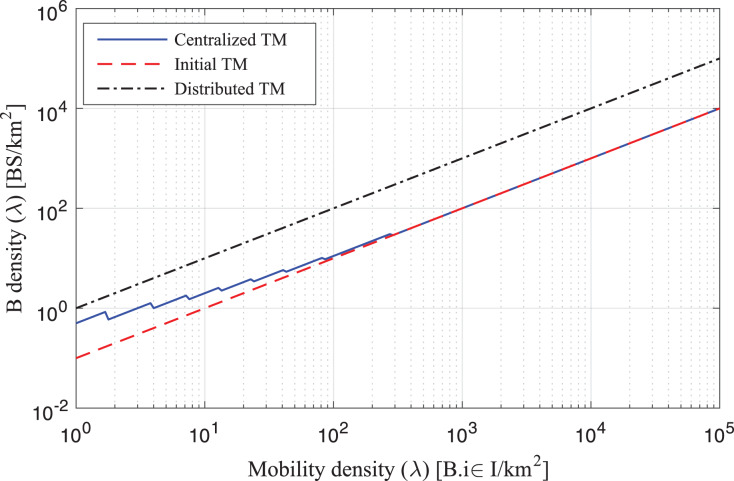
Federated learning delay.

**Figure 6:**
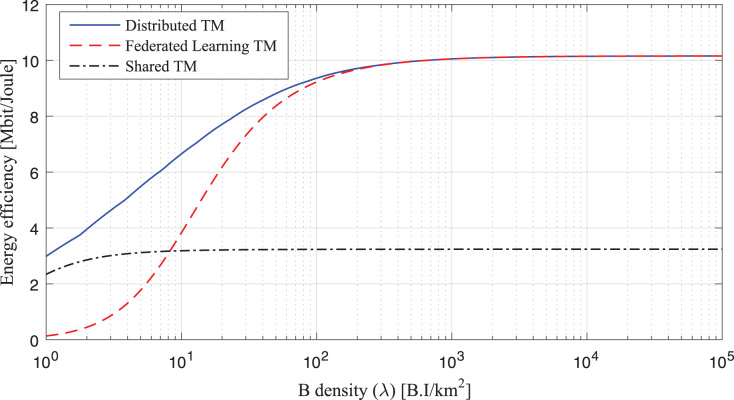
Energy consumption during distributed federated learning.

The study analyses the spike of workload requests to base stations as per B density *λ* (BS/km^2^) Per Requested Workloads in the simulation as shown in Fig. 3. The density *λ* offers the arrival requests of workloads to the fog-enable base stations during different intervals. At the same time, the process consumes *B*_*Energy*__1_, *B*_*Energy*__1,2_ unit per joule decision of managing workloads with the IRS Unit. The study analyses the spike of workload requests to base stations as per B different density’s *λ* (BS/km^2^) Per Requested Workloads in the simulation as shown in Fig. 4. The density *λ* offers the arrival requests of workloads to the fog-enable base stations during different intervals. In contrast, the process consumes *B*_*Energy*__1_, *B*_*Energy*__1,2_ unit per joule decision of managing workloads with the IRS Unit. It shows different density requests have required different energy level to process the recommendations in the cellular network. Figures 5 and 6 denotes the mobility and base-station to base-stations FL-IRS outperforms as compared existing state of art IRS method. Figures 5 and 6 FL-IRS unit can achieve optimal results in the existing reconfigurable cellular stations with the specific ranges with different density workloads level in the system.

### Result discussion part

This part shows the performances of different schemes in the IRS that enable the unit to the designed system for the healthcare applications.

#### Energy consumption between sensors and base-stations

**Figure 7:**
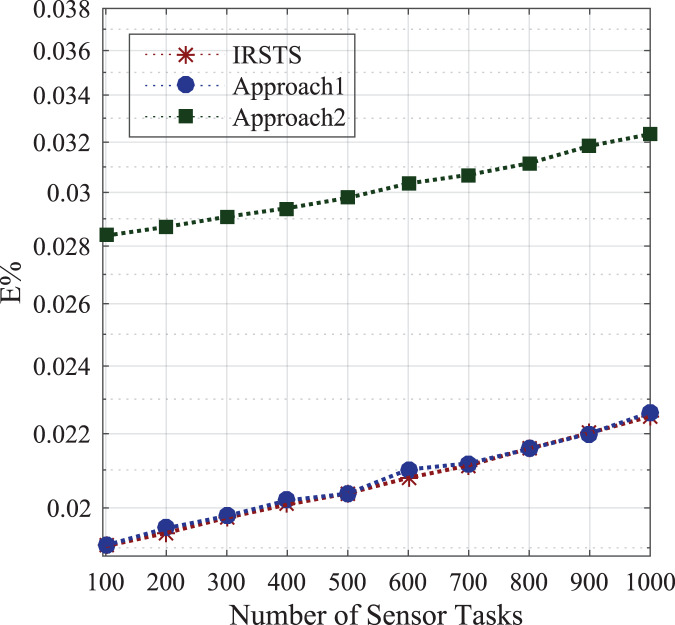
Energy consumption between sensors and base-stations.

**Figure 8:**
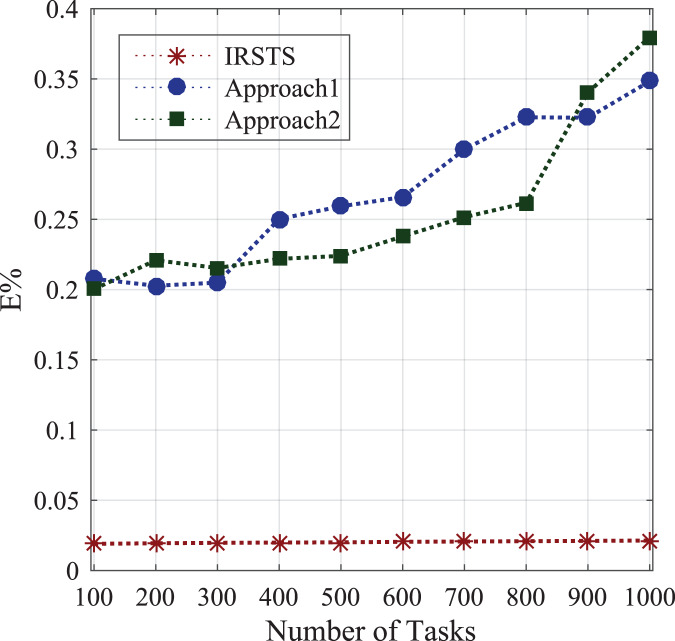
Energy consumption between base-station to base-station.

**Figure 9:**
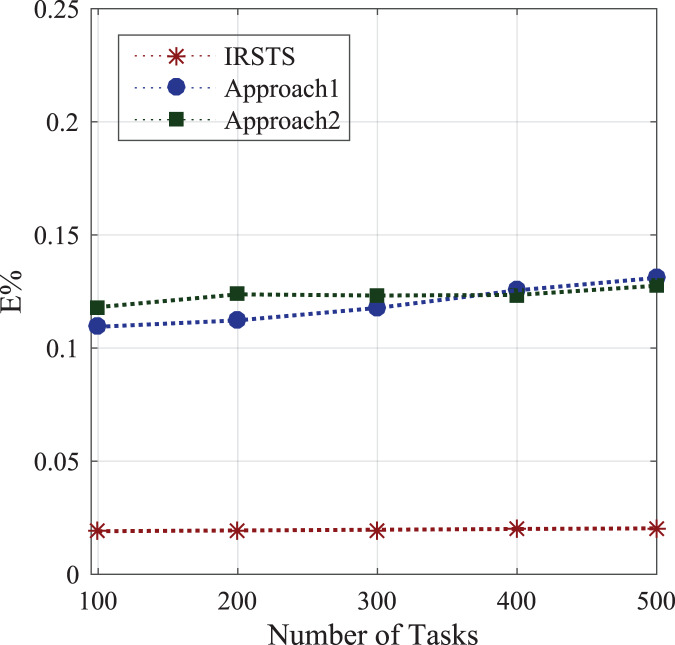
Energy consumption between sensors and base-stations during high requests.

**Figure 10:**
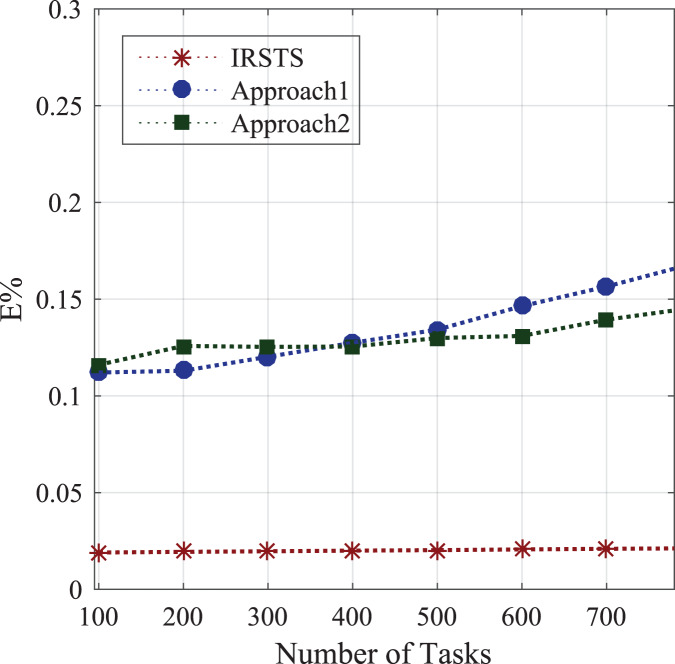
Energy consumption between sensors and base-stations during peak hours.

Initially, the study determines the energy-efficient performances between requested sensors workload and fog-enable base-stations as defined in Eq. (1). Figure 7 shows the federated learning aware base-station with IRS can make the optimal decision compared to the state of the art IRS method for the cellular system. The proposed system IRSTS determines the density request traffics at the runtime and learning them with efficient-energy efficiency and delay performances as shown in Figs. 7 and 8 during initial and base-station to base-station as defined in Eq. (2). Figures 9 and 10 elaborated the energy-performances of all cases as defined in Figs. 2A and 2B with the federated learning aware IRS unit with fog-enable base-stations. The existing approaches exploited IRS with the cellular base-stations without learning and with the fixed number of requests in the different intervals as did in Approach1 and Approach2. However, IRSTS schemes in the proposed distributed the teaching in other parts as FCA can decide with lower power consumption during initial energy, base-station to base-station energy and mobility energy. In this way, the efficient routing path for healthcare applications can deploy and optimize the energy objective *E*% of the study efficiently.

**Figure 11:**
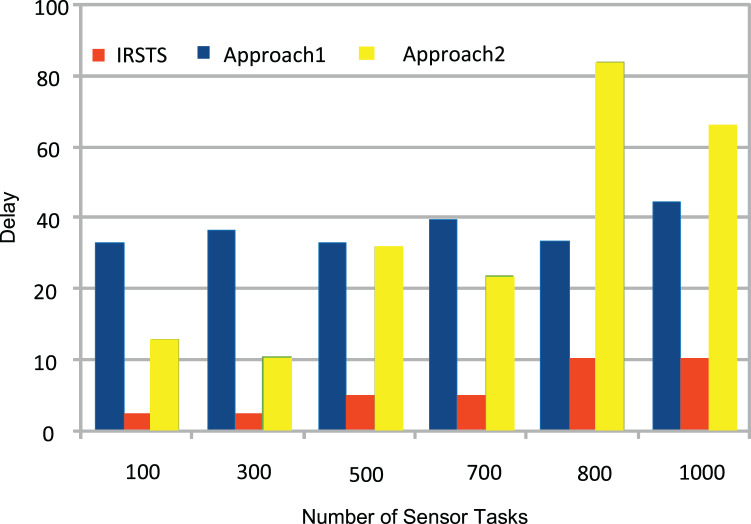
Offloading between sensors and base-stations.

**Figure 12:**
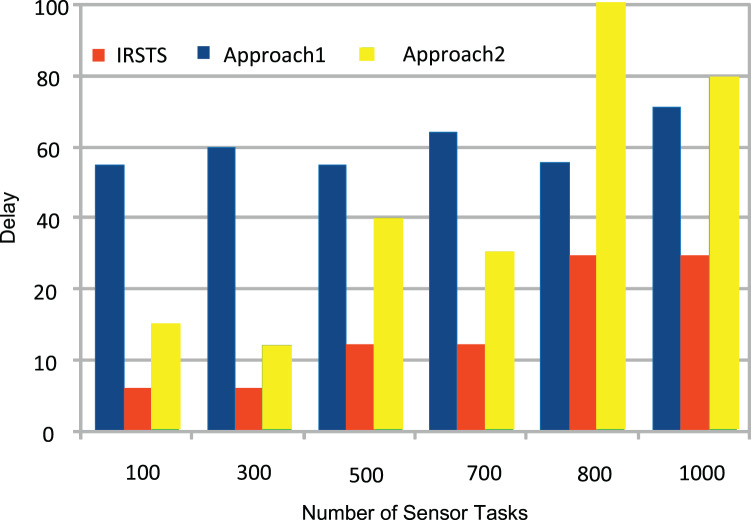
Execution delay at nodes.

Figures 11 and 12 shown the performances of IRSTS as compared to Approach1 and Approach2 with delay objective for healthcare workloads in the system. It is cleared from results figures, the IRSTS outperformed all existing IRS enable cellular system with their approaches. The main reason is that, IRSTS divided the overall learning of the system in different layers as shown in Figs. 2A and 2B. By this way, the delay of centralized existing IRS enable fog-cloud can improved with the proposed schemes in the system.

### Finding of the proposed scheme

The main finding of the study is to achieve distributed learning of nodes in terms of latency, efficiency, and energy for all applications in the system. In this way, many things can improve, for instance, the decision capability of each node and train sensory data on local nodes and shared to the global node. It is different from the existing machine learning model where data train on a single node and shared to another node; however, each time node access from centralized node and it has a high cost of overhead, energy, and lateness to the applications. In the simulation, the result discussion parts showed the proposed idea outperformed all existing techniques in terms of objectives for all applications.

### Limitation of the work

The existing work improved the centralized learning model in terms of energy and latency with the decentralized proposed schemes of IRS technology. The current machine learning-enabled centralized learning model incurred with the heavyweight overhead and learning rate is too slow. The main reason is that all models of different applications are trained on a single centralized node. Therefore, the distributed learning model based on federated distributed learning is trained data on local devices and shared to the fog nodes. In this way, resources, energy, and training time in terms of latency are minimized for healthcare applications. However, there are many limitations of the current study. For instance, the current does not consider the mobility services of the application. The present work does not analyze the security and node validation between the shared model and local model of the data in the system. The cost-efficient scheduling is to be investigated more in terms of the failure of workload in the system.

## Conclusion and Future Work

The federated learning aware Intelligent Reconfigurable Surface Task Scheduling schemes (FL-IRSTS) algorithm is proposed in this paper to achieve high-speed communication with energy and delay efficient offloading and scheduling. The trained model will decide the IRSTS configuration that best meets the goals in terms of communication rate. For each workload, multiple local models trained with the local healthcare fog-cloud network using federated learning (FL) to generate a global model. Then, each trained model shared its initial configuration with the global model for the next training round. Each application’s healthcare data is handled and processed locally during the training process. Simulation results show that the proposed algorithm’s achievable rate output can effectively approach centralized machine learning (ML) while meeting the study’s energy and delay objectives.

In the future, the study will consider the blockchain-enabled FL-IRSTS system for Industrial Internet of Things applications in the distributed fog-cloud network.

## Additional Information and Declarations

### Data Availability

The following information was supplied regarding data availability: The data is available at figshare: kadry, seifedine (2021): Federated learning enables intelligent-Source-Code-2021.zip. figshare. Dataset. https://doi.org/10.6084/m9.figshare.16641478.v1.

## References

[ref-1] Chen X, Hu J, Chen Z, Lin B, Xiong N, Min G (2021). A reinforcement learning empowered feedback control system for industrial internet of things. IEEE Transactions on Industrial Informatics.

[ref-2] Chen P-HC, Liu Y, Peng L (2019). Healthcare dataset in machine learning. Nature Materials.

[ref-3] Hosseinalipour S, Brinton CG, Aggarwal V, Dai H, Chiang M (2020). From federated to fog learning: distributed machine learning over heterogeneous wireless networks. IEEE Communications Magazine.

[ref-4] Kousiouris G, Tsarsitalidis S, Psomakelis E, Koloniaris S, Bardaki C, Tserpes K, Nikolaidou M, Anagnostopoulos D (2019). A microservice-based framework for integrating IoT management platforms, semantic and AI services for supply chain management. ICT Express.

[ref-5] Lakhan A, Ahmad M, Bilal M, Jolfaei A, Mehmood RM (2021a). Mobility aware blockchain enabled offloading and scheduling in vehicular fog cloud computing. IEEE Transactions on Intelligent Transportation Systems.

[ref-6] Lakhan A, Dootio MA, Groenli TM, Sodhro AH, Khokhar MS (2021b). Multi-layer latency aware workload assignment of e-transport IoT applications in mobile sensors cloudlet cloud networks. Electronics.

[ref-7] Lakhan A, Li X (2019). Content aware task scheduling framework for mobile workflow applications in heterogeneous mobile-edge-cloud paradigms: catsa framework.

[ref-8] Lakhan A, Li X (2020). Transient fault aware application partitioning computational offloading algorithm in microservices based mobile cloudlet networks. Computing.

[ref-9] Lakhan A, Mohammed MA, Rashid AN, Kadry S, Panityakul T, Abdulkareem KH, Thinnukool O (2021c). Smart-contract aware ethereum and client-fog-cloud healthcare system. Sensors.

[ref-10] Lakhan A, Xiaoping L (2018). Energy aware dynamic workflow application partitioning and task scheduling in heterogeneous mobile cloud network.

[ref-11] Mujeeb-ur Rehman AL, Hussain Z, Khoso FH, Arain AA (2021). Cyber security intelligence and ethereum blockchain technology for e-commerce. International Journal of Emerging Trends in Engineering Research.

[ref-12] Qu Y, Gao L, Luan TH, Xiang Y, Yu S, Li B, Zheng G (2020). Decentralized privacy using blockchain-enabled federated learning in fog computing. IEEE Internet of Things Journal.

[ref-13] Saha R, Misra S, Deb PK (2020). FogFL: Fog assisted federated learning for resource-constrained IoT devices. IEEE Internet of Things Journal.

[ref-14] Sharma PK, Park JH, Cho K (2020). Blockchain and federated learning-based distributed computing defence framework for sustainable society. Sustainable Cities and Society.

[ref-15] Zhao Z, Feng C, Yang HH, Luo X (2020). Federated-learning-enabled intelligent fog radio access networks: fundamental theory, key techniques, and future trends. IEEE Wireless Communications.

[ref-16] Zhou C, Fu A, Yu S, Yang W, Wang H, Zhang Y (2020). Privacy-preserving federated learning in fog computing. IEEE Internet of Things Journal.

